# Synthesis and assembly of colloidal cuboids with tunable shape biaxiality

**DOI:** 10.1038/s41467-018-06975-8

**Published:** 2018-10-30

**Authors:** Yang Yang, Guangdong Chen, Srinivas Thanneeru, Jie He, Kun Liu, Zhihong Nie

**Affiliations:** 1 Department of Chemistry and Biochemistry, University of Maryland, College Park, MD 20742 USA; 2 State Key Laboratory of Supramolecular Structure and Materials, College of Chemistry, Jilin University, Changchun, 130012 China; 3 Department of Chemistry, Institute of Materials Sciences, University of Connecticut, Storrs, CT 06269 USA

## Abstract

The design and assembly of monodisperse colloidal particles not only advances the development of functional materials, but also provides colloidal model systems for understanding phase behaviors of molecules. This communication describes the gram-scale synthesis of highly uniform colloidal cuboids with tunable dimension and shape biaxiality and their molecular mesogen-like assembly into various mesophasic structures in pristine purity. The synthesis relies on the nanoemulsion-guided generation of ammonium sulfate crystals that template the subsequent silica coating. The shape of the cuboidal particles can be tuned from square platelike, to biaxial boardlike, and to rodlike by independently controlling the length, width and thickness of the particles. We demonstrated the assembly of the cuboidal colloids into highly pure mesoscopic liquid crystal phases, including smectic A, biaxial smectic A, crystal B, discotic, and columnar phases, as well as established a correlation between mesophasic formation and colloidal biaxiality in experiments.

## Abstract (web summary)

Monodisperse biaxial colloidal particles are used as model systems to understand the phase behavior of biaxial molecules but high-quality biaxial colloids are limited. Here the authors describe gram-scale synthesis of uniform biaxial colloidal cuboids and their assembly into mesophasic structures.

## Introduction

Liquid crystalline materials are ubiquitous in nature and our everyday life. Other than molecular liquid crystals (LCs), suspensions of some shaped colloidal particles (e.g., inorganic or biological rods, bowls, and platelets) are known to exhibit entropy-driven liquid crystalline phases, such as nematic, smectic, bowlic, discotic (N_−_), and columnar (Col) phases^1–3^. The nature and transition of these mesophasic structures are strongly dependent on the geometry and uniformity of colloidal particles^4–8^. Because of their fluidity and mesoscopic ordering in different forms, colloid-based liquid crystalline materials show great potential in applications, ranging from sensors to display devices and to metamaterials^9,10^. Compared with molecular LC systems, LC phases of colloidal particles exhibit distinctive features and they are (i) all lyotropic; (ii) driven by entropy alone; (iii) susceptible to polydispersity of constituent colloids; and (iv) inexpensive, thermally stable, and more susceptible to external fields^11^. Moreover, they can be quantitatively studied on the single particle level owing to the unique dimension (from hundreds of nanometers to micrometer scale) of colloids. Thus, it is of great fundamental and technological importance in designing novel colloidal particles and understanding their LC behaviors^12–15^.

The past decades have witnessed rapid advances in the synthesis of anisotropic colloidal particles with controlled shapes and crystallographic facets by using structure-directing agents^16^, including small molecules (e.g., nitrogen dioxide)^17^, ions (e.g., citrate and bromide ions)^18–20^, surfactants (e.g., hexadecyltrimethylammonium bromide)^21^, polymers (e.g., polyvinylpyrrolidone, PVP)^22^, and biomolecules (e.g., peptides)^23^. Biaxial colloidal particles are of great interest, due to their ability to form biaxial phases with two optic axes (where each symmetry axis has a preferred direction of alignment), which are promising candidates for LC displays^24,25^. Shape biaxiality of biaxial colloids (e.g., biaxial ellipsoids, bent-core particles, and boardlike particles) can promote the formation of the biaxial ordering. In spite of the fact that biaxial LC molecules represent a large class of mesogens for materials discovery^24^, experimental studies of biaxial colloidal systems have been rare^26,27^. This is largely due to the limited access to high-quality colloids with the inherent biaxial shape. Currently available biaxial colloids include bent silica rod^28^, goethite^29^, hectorite^30^, and lead carbonate boardlike particles^31^ with unique *D*_2h_ symmetry^32–34^, of which goethite particles are mostly studied. These goethite colloids, however, show very broad polydispersity (up to 55%) in size and shape. The assembly of such colloids leads to the coexistence of multiple phases and genetic defects (rather than single pristine phases), which hinders a deep understanding of their LC behaviors and the discovery of potential applications of resulting materials^35^.

This communication presents the gram-scale synthesis of highly monodisperse colloidal cuboids with tunable dimension and shape biaxiality and their molecular mesogen-like assembly into various mesophasic structures in pristine purity. This synthetic method relies on the nanoemulsion-guided formation of well-defined ammonium sulfate ((NH_4_)_2_SO_4_) crystals that template the subsequent hydrolysis of tetraethoxysilane (TEOS) to produce uniform cuboids in a one-pot reaction with iron (III) cation (Fe^3+^), PVP, and sodium citrate (SC) as structure-directing agents. The resulting cuboidal particles exhibit a narrow size distribution (polydispersities <5%) in all three dimensions. The shape of the particles can be tuned from square platelike, to biaxial boardlike, and to rodlike by independently controlling the length (*L*), width (*W*), and thickness (*T*) of the particles. These colloidal cuboids resemble organic boardlike mesogens to assemble into a rich variety of LC phases, including uniaxial smectic A (SmAu), biaxial smectic A (SmAb), crystal B, N_−_, and Col phases. The formation of pristine, pure mesophases (rather than coexistence of multi-phases in one system) enables us to experimentally reveal the critical role of shape biaxiality in cuboidal colloid assembly. A phase diagram is constructed to identify the stable phases and describe the phase transition of the assembly system. This work provides a class of colloidal building blocks for fabricating biaxial structural materials with potential applications in fast electro-optical switching devices. Moreover, it offers a mesoscopic model of LC molecules for understanding molecular assembly.

## Results

### Synthesis of colloidal cuboids

**Fig. 1:**
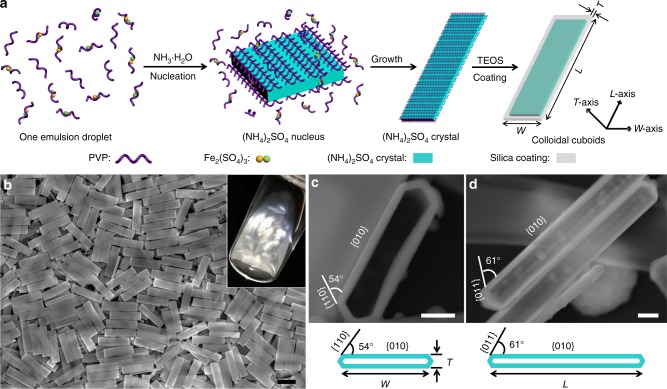
Schematic illustration of the synthesis and structure of colloidal cuboids. **a** Schematics illustrating the synthesis of colloidal cuboids based on (NH_4_)_2_SO_4_ crystal-templated hydrolysis of silica precursors. **b** SEM image of representative monodisperse cuboidal particles (*L* = 3600 nm, *W* = 870 nm, *T* = 220 nm). Inset photo shows the birefringence of a suspension of 0.2 wt% colloids in ethanol upon shaking (taken between cross-polarizers). **c**, **d** SEM images of cuboidal particles after removal of (NH_4_)_2_SO_4_ core: **c** top view of a fractured cuboid with an open silica shell and **d** side view of intact cuboids with closed silica shell. Bottom schematics illustrate the corresponding facets of (NH_4_)_2_SO_4_ crystal. Scale bars, 2 μm in (**b**) and 200 nm in (**c**, **d**)

The cuboidal colloids were synthesized by in situ generation and growth of well-defined (NH_4_)_2_SO_4_ crystals initiated within discrete nanoscale reactors of water emulsions containing directing agents, Fe^3+^, PVP, and SC, and subsequent coating of silica on the surface of crystal templates. In a typical synthesis, an aqueous solution containing ethanol, iron (III) sulfate (Fe_2_(SO_4_)_3_), and SC was first emulsified in *n*-pentanol dissolved with PVP to generate nanosized water-in-oil emulsions (Fig. 1a and see Methods for experimental details)^28,36,37^. Ammonia (NH_3_) was then introduced into the mixture, followed by or together with the addition of silica precursor, TEOS. The rapid reaction (in ~30–60 s) of NH_3_ with Fe_2_(SO_4_)_3_ within the emulsion droplets produced highly uniform (NH_4_)_2_SO_4_ crystals with well-defined boardlike shape. Dynamic light scattering (DLS) analysis shows that the emulsion droplets were around 160 ± 6.4 nm in diameter (Supplementary Fig. 1). We presume that the formation of monodisperse (NH_4_)_2_SO_4_ crystals is attributed to the high uniformity of the emulsions that serve as nanoreactors and determine the amount of salt precursors available for individual crystals. The subsequent hydrolysis and condensation of TEOS—which was catalyzed by residue of NH_3_—on the surface of crystals yielded monodisperse cuboidal colloids (polydispersities below 5% in all three dimensions, defined as the standard deviation *σ* of *L*, *W*, and *T* divided by the mean values of *L*, *W*, and *T*, respectively) composed of (NH_4_)_2_SO_4_ core and silica shell (Fig. 1b and the particle composition will be further discussed in detail later). The coating of the silica shell also prevented the overgrowth and size broadening of the crystals. The critical role of water emulsions in the synthesis was confirmed by our two control experiments: highly polydispersed colloids with limited control over size and shape were produced, when (NH_4_)_2_SO_4_ salt was directly added in the reaction without in situ generation of (NH_4_)_2_SO_4_ or when the synthesis was conducted in bulk water instead of water nanoemulsions (Supplementary Fig. 2). The cuboidal colloids can be well dispersed in ethanol and stable for at least 1 year (Supplementary Fig. 3 and Supplementary Fig. 4). Such a suspension with 0.2 wt% of cuboids showed a remarkable flow birefringence under gentle shaking-induced shear, which disappeared within several seconds at rest (Fig. 1b)^38^.

### Characterization of cuboidal structure and composition

It is noted that the cuboidal colloids preserve the shape and even crystal facets of crystal templates. The (NH_4_)_2_SO_4_ cores can be readily removed by washing the particles with water, leaving hollow silica shells with boardlike shape (Fig. 1c, d and Supplementary Fig. 5). Scanning electron microscopy (SEM) images show that the silica shells were composed of {011}, {110}, and {010} facets originated from the (NH_4_)_2_SO_4_ crystals. The angles between two adjacent facets (i.e., {010}/{110} and {010}/{011} pairs) of the hollow shell were found to be ~54° and ~61°, respectively.

**Fig. 2:**
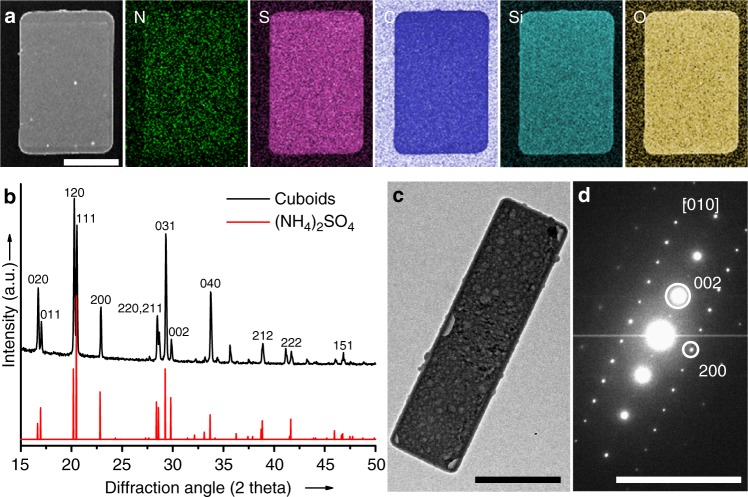
Characterization of the structure and composition of colloidal cuboids. **a** Annular dark field TEM image and TEM-EDS maps of a cuboidal particle (*L* = 5100 nm, *W* = 3400 nm, *T* = 80 nm). **b** XRD pattern, **c** TEM image, and **d** corresponding selected area electron diffraction pattern of a cuboid (*L* = 3200 nm, *W* = 880 nm, *T* = 180 nm). Scale bars, 2 μm in (**a**), 1 μm in (**c**), and 10 nm^−1^ in (**d**)

The structure and composition of cuboids were characterized by various techniques. An annular dark-field transmission electron microscope (TEM) image and energy-dispersive X-ray spectrometer (EDS) maps of a typical particle indicate a uniform distribution of N, S, C, Si, and O, but no presence of Fe across the whole particle (Fig. 2a and Supplementary Fig. 6). Fourier transform infrared spectroscopy (FT-IR) spectra of cuboids before and after removing the cores also suggest the presence of (NH_4_)_2_SO_4_ and silica components in particles (Supplementary Fig. 7). The formation of pure (NH_4_)_2_SO_4_ crystal was further confirmed by powder X-ray diffraction (XRD) pattern (Fig. 2b). The diffraction peaks matched well with the Powder Diffraction Standards file no. 00-040-0660 of orthorhombic (NH_4_)_2_SO_4_. It is worth noting that the peak intensity ratios of the (020)/(111) and (040)/(111) are significantly higher than those of bulk crystals, indicating that the crystalline cores of cuboids are abundant with {010} facets. Moreover, the symmetry of TEM electron diffraction pattern further confirmed that the core of particle is a single crystal bounded mainly by {010} planes, which is consistent with the XRD results (Fig. 2c, d and Supplementary Fig. 8).

### Control over cuboidal morphology and dimensions

**Fig. 3:**
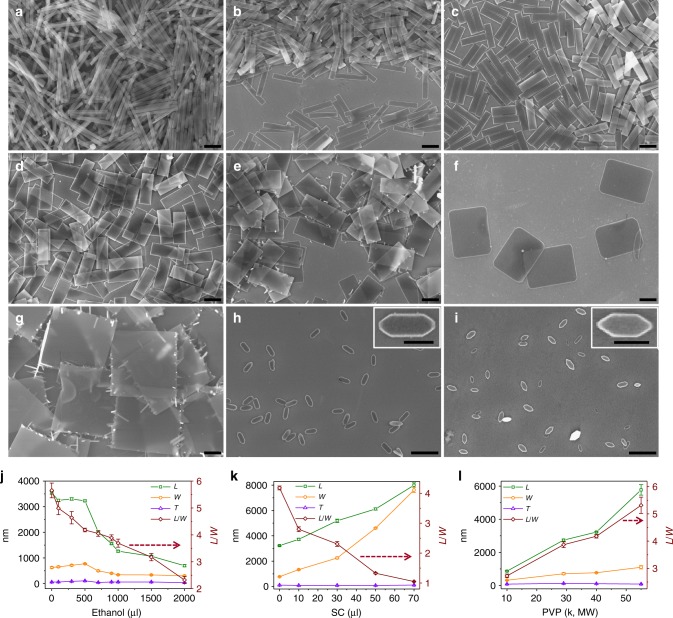
Representative SEM images of colloidal cuboids with different shapes. Cuboidal particles in **c** were synthesized with ethanol (500 μl), Fe_2_(SO_4_)_3_ (40 mg ml^−1^, 140 μl), NH_3_ (60 μl), SC (0 μl), and TEOS (50 μl). Using synthetic condition for **c** as reference, the adjustments for synthesizing other cuboidal particles were: **a** NH_3_ (200 μl), **b** ethanol (0 μl), **d** SC (0.18 M, 10 μl), **e** SC (0.18 M, 30 μl), **f** SC (0.18 M, 50 μl), **g** SC (0.18 M, 70 μl), **h** ethanol (1800 μl), and **i** ethanol (2000 μl), while other parameters were the same. Scale bars, 2 μm in (**a**–**i**) and 500 nm in insets of (**h**, **i**). **j**–**l** The dependency of colloidal geometry on the amount of (**j**) ethanol, **k** SC, and **l** MW of PVP. Error bars in (**j**–**l**) represent standard deviations by measuring 200 samples for each point

The morphology and dimensions of cuboidal colloids can be controlled by varying reaction conditions, such as molecular weight (MW) of PVP, reaction temperature, and the amount of NH_3_, Fe_2_(SO_4_)_3_, SC, ethanol, and TEOS (Fig. 3 and Supplementary Figs. 9–14). The *L*/*W* of monodisperse cuboids increased from 3 up to 13 with increasing the amount of NH_3_ from 20 to 200 μl (Fig. 3a and Supplementary Fig. 9). When the amount of NH_3_ was out of this range, the synthesis produced cuboids with broad size distribution. In the absence of ethanol, the *L*/*W* of cuboids could be adjusted up to 6, due to the lengthening of *L* (Fig. 3b). When ethanol was added, both the dimension and *L*/*W* of cuboids gradually decreased with the increase in the ethanol amount (Fig. 3j and Supplementary Fig. 11). Further increase in ethanol amount (>1500 μl) led to the colloidal shape transformation from rectangle to elongated hexagonal boardlike (Fig. 3h, i). On the contrary, both *L* and *W* increased with increasing the added amount of SC; and this process was accompanied with a shape transformation of cuboids from rectangle to square boardlike (Fig. 3c–g, k). When excessive SC was added, the surfaces of cuboids become rough, due to the growth of nanowhiskers at edges (Fig. 3g)^36^. Both the size and *L*/*W* were found to increase with increasing the MW of PVP (Fig. 3l and Supplementary Fig. 12). Moreover, the thickness of cuboids could be controlled in a range of 30–200 nm by tuning the amount of TEOS and the growth time in the one pot synthesis. Thicker cuboids (*T* up to 700–900 nm) could be obtained by post-coating of silica on the particles, while not losing their boardlike shape.

### Key factors affecting cuboidal shape

Three key factors including Fe^3+^ cation, PVP, and SC were found to be indispensable for the formation of colloidal cuboids with a defined shape. The presence of Fe^3+^ cation was found to be critical for the formation of shaped (NH_4_)_2_SO_4_ crystals. Other than Fe^3+^, Fe^2+^ and Ce^3+^ cations could also lead the formation of shaped crystals, thanks to their preferential strong interaction with the {010} crystal facet relative to others (Supplementary Fig. 15). However, other cations, such as Na^+^, Mg^2+^, Ca^2+^, Mn^2+^, Ni^2+^, Cu^2+^, and Cd^2+^, could not induce the cuboidal growth of (NH_4_)_2_SO_4_, probably due to the low valence and thereby weak interaction with crystal surfaces^19,20^. Moreover, both PVP and SC are also known as structure-directing agents that can selectively interact with various crystallographic planes^39^. Take noble metal nanocrystals as an example, PVP binds preferentially to {100} facets, while SC binds more strongly to {111} than {100} facets. Given the fact that rectangle cuboidal shape can be generated only with the presence of PVP, it is believed that PVP selectively absorbs on the {010} facets and reduces the growth rate largely along the 〈010〉 direction and slightly along the 〈100〉 direction, leading to the formation of boardlike rectangle crystals^40^. In contrast, SC may preferentially bind to {011} and {110} facets more strongly than PVP, thus increasing the relative growth rates along both 〈100〉 and 〈001〉 directions. This phenomenon was evidenced by our observation that the introduction of SC led to an increase in both *L* and *W* of cuboids as well as the shape transformation of cuboids from rectangle to square boardlike. We, therefore, conclude that Fe^3+^, PVP, and SC serve as directing agents in the formation of shaped crystals and hence cuboids with tunable geometries.

### LC phases assembled from cuboids with various geometries

**Fig. 4:**
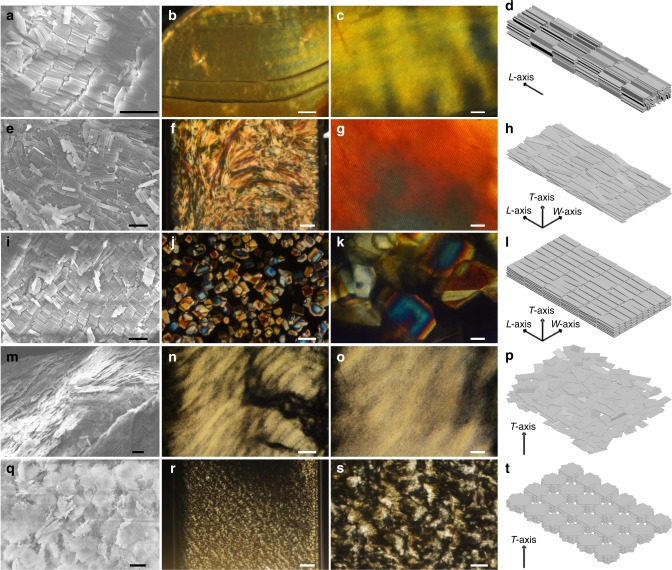
LC phases of cuboidal colloids with different geometries. Representative (**a**, **e**, **i**, **m**, **q**) SEM images, corresponding large-area (**b**, **f**, **j**, **n**, **r**) and enlarged (**c**, **g**, **k**, **o**, **s**) POM images, and schematic illustrations (**d**, **h**, **l**, **p**, **t**) of LC phases assembled from different cuboidal colloids in planar capillary: SmAu phase, cuboids of *L* = 2350 nm, *W* = 360 nm, *T* = 125 nm (**a**–**d**), SmAb phase, cuboids of *L* = 3270 nm, *W* = 830 nm, *T* = 78 nm (**e**–**h**), crystal B phase, cuboids of *L* = 2640 nm, *W* = 750 nm, *T* = 100 nm (**i**–**l**), N_−_ phase, cuboids of *L* = 4080 nm, *W* = 1530 nm, *T* = 70 nm (**m**–**p**), Col phase, cuboids of *L* = 3720 nm, *W* = 3040 nm, *T* = 70 nm (**q**–**t**). Scale bars, 5 μm in (**a**, **e**, **i**, **m**, **q**), 100 μm in (**b**, **j**, **n**), 200 μm in (**f**, **r**), 20 μm in (**c**, **g**, **k**, **o**), and 50 μm in (**s**)

Remarkably, monodisperse colloidal cuboids with various shapes resemble boardlike mesogens to assemble into various mesoscopic LC phases via slow sedimentation in solvent (Fig. 4). It should be emphasized that owing to the uniformity of colloids, the coexistence of multiple phases was not observed in one system. With the particle shape changing from rodlike geometry, to biaxial boardlike, and to square platelike, the LC phases transited from SmAu, to SmAb, to N_−_, and finally to Col phases via sedimentation of colloids from their ethanol suspensions. In particular, when dimethyl sulfoxide (DMSO), which has a density closer to silica than ethanol, was used, the relatively slow sedimentation of particles led to the formation of highly ordered crystal B phase, thanks to the extremely high uniformity of colloidal cuboids.

For the rodlike cuboids, uniaxial SmAu phase structure was produced, as indicated by SEM and polarized optical microscopy (POM) characterization (Fig. 4a–d and Supplementary Fig. 16). In the assembled phase, the *L*-axis of cuboids is perpendicular to the smectic layer and their *W*- and *T*-axes randomly orient within the layers (Fig. 4a). The formation of uniaxial SmAu phase was confirmed by characteristic focal conic textures and obvious strips corresponding to the layered structure of SmAu phase in regular and enlarged POM images (Fig. 4b, c). In comparison, cuboids with distinct shape biaxiality spontaneously organized into SmAb phase in ethanol (Fig. 4e–h and Supplementary Fig. 17). Cuboids in the biaxial phase not only spontaneously aligned their *L*-axis parallel to the layer normal, but also oriented their *W*- and *T*-axes in the layer plane perpendicular to the layer normal (Fig. 4e). This phase of cuboids exhibited two macroscopic optical axes, thus displaying two-brush disclinations in the POM textures, in contrast to the four-brush disclinations in the textures of SmAu phase (Fig. 4f). Typical layered textures were also observed in the enlarged POM image (Fig. 4g). Interestingly, in DMSO, the slow sedimentation of the same colloids produced crystal B phase rather than SmAb phase (Fig. 4i–l and Supplementary Fig. 18). In this phase, cuboidal particles closely packed to form ordered structures, where each axis of cuboids possesses a preferred direction of alignment (Fig. 4i). The resulting microcrystals showed random crystal orientation in flat capillaries (Fig. 4j, k). The crystal behavior of cuboidal colloids is similar to that of boardlike small molecular mesogens^41^ and monodisperse rodlike tobacco mosaic viruses^42^ reported previously. As the cuboids became even wider, they were stabilized in the N_−_ phase (Fig. 4m–p and Supplementary Fig. 19). In this case, flat faces of cuboids are parallel to each other, while their short axes are aligned along the nematic director (Fig. 4m). POM images displayed a typical Schlieren texture of N_−_ phase (Fig. 4n, o). For square platelike cuboids, they stacked in columns that organized into Col phase, as indicated by the broken fan-shaped texture (Fig. 4q–t and Supplementary Fig. 20). The vertical stripes in patterns correspond to the columns, as shown in the enlarged POM image (Fig. 4s).

### Phase diagram and shape biaxiality effect

**Fig. 5:**
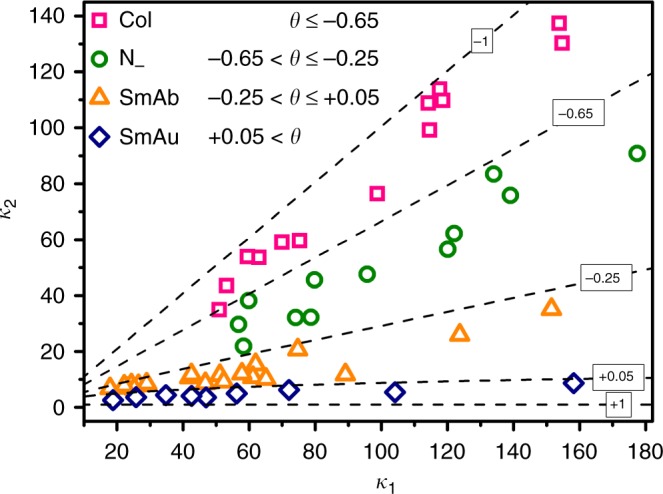
Colloidal shape biaxiality effect on the phase diagram. Phase diagram of cuboidal particles as a function of *κ*_1_ and *κ*_2_. The dashed lines correspond to different *θ* values as shown on the lines

We systematically investigated the effect of colloidal geometry on the LC behaviors of cuboidal colloids and mapped the five mesophases in a phase diagram (Fig. 5). Moreover, the formation of mesophases was further rationalized in terms of the particle shape biaxiality *θ* which can be calculated as^33^:

1 $$\theta \equiv \left( {\kappa _1 - 1} \right)^{ - 1}\left( {\frac{{\kappa _1}}{{\kappa _2}} - \kappa _2} \right)$$

where two aspect ratios are *κ*_1_ = *L*/*T* and *κ*_2_ = *W*/*T*, respectively. *θ* varies from −1 (uniaxial platelike geometry), via 0 (i.e., *L*/*W* = *W*/*T*, perfect biaxiality), to 1 (uniaxial rodlike geometry)^34^. Specifically, rodlike colloids with less noticeable biaxial shape (*θ* > 0.05) were stabilized in SmAu phase, as they can rotate freely along the *L*-axis because of the relatively small energy barriers for their rotation within layers. Biaxial boardlike cuboids with *θ* around 0 preferred to form SmAb phase in ethanol and crystal B phase in DMSO. The formation of crystal B phase is ascribed to the high uniformity of colloidal particles, comparable to small molecular mesogens. As *θ* decreased to the range of −0.65 < *θ* ≤ −0.25, the biaxial shape was not sufficient to drive the formation of the SmAb phase. Instead, the cuboidal colloids were accommodated in N_−_ phase. At *θ* ≤ −0.65, the particles turned into square platelike, resulting in the phase transition to Col phase. Overall, these results manifest that the rich mesophase polymorphism and the phase sequence of cuboidal colloids are strongly dependent on their shape biaxiality and sedimentation rate of colloids during assembly.

## Discussion

In conclusion, we have developed a simple and scalable approach to synthesize monodisperse colloidal cuboids with tunable shape geometries and dimensions. The use of water emulsions as discrete nanoscale reactors to guide the growth of crystals and subsequent templated coating of silica is unique, enabling control over the physical features of cuboids. We further systematically studied the phase behavior of colloidal cuboids and determined the critical role of shape biaxiality on the formation of mesoscopic ordering phases. Remarkably, the assembly of cuboidal colloids produces pure SmAu, SmAb, crystal B, N_−_, and Col phases. This work offers insights into the phase behavior of boardlike molecular or colloidal mesogens. Moreover, it provides a library of colloidal building blocks for the fabrication of functional materials with broad applications in such as photonic crystals (Supplementary Fig. 21), sensors, and display devices.

## Methods

### Synthesis of colloidal cuboids

Typically, a solution containing 500 mg of PVP (MW = 40,000, Sigma-Aldrich) and 5.0 ml of *n*-pentanol (99%, Sigma-Aldrich) was prepared in a 10 ml glass vial. A 140 μl aqueous solution of 40 mg ml^−1^ Fe_2_(SO_4_)_3_ (Sigma-Aldrich) and a 500 μl of anhydrous ethanol (Pharmco-Aaper) were added into the glass vial and mixed thoroughly under vortex stirrer. The mixed solution was kept standing for 1 min to release gas bubbles. Then, a 60 μl aqueous solution of NH_3_ (28 wt%, Sigma-Aldrich) and a 50 μl of TEOS (98%, Sigma-Aldrich) were added into the solution in sequence. After each addition, the mixture was gently shaken for 30 s. The mixture was incubated at 25 °C for 4 h to allow the reaction complete.

### Purification

After the completion of the reaction, the solution containing synthesized colloidal cuboids was then centrifuged at 6000 rpm for 10 min. The supernatant was removed and the particles were washed three times with ethanol at 3000 rpm. Finally, the particles were centrifuged at 1000 rpm for 10 min to remove lightweight iron hydroxide small particle impurities.

### Further silica coating on colloidal cuboids

Typically, cuboidal colloids (10 mg) were dispersed in ethanol (5 ml) in a 10 ml glass vial. Deionized water (18.2 MΩ, 500 μl) and NH_3_ aqueous solution (28 wt%, 50 μl) were added and mixed. Then TEOS (50 μl) was added dropwise under stir.

### Core remove

Purified cuboids were dispersed in water upon vortex. The solution was then centrifuged at 4000 rpm for 10 min, and the cores are fully removed after three times wash with water.

### Assembly of colloidal cuboids into LCs

A dispersion of colloidal cuboids in ethanol or DMSO (99.9%, Fisher Chemical) was filled into a one-end-sealed glass tube with an outer diameter of 1.9 mm, inner diameter of 1.3 mm, and length of 100 mm (Stuart) and a rectangular capillary tube with a dimension of 0.2 × 2 × 50 mm (VitroCom). The other end of the tube was then heated and sealed by melting the glass. The tube was placed vertically to allow the slow sedimentation of colloidal particles for 1 month. Afterwards, the sediment of assembled colloids in the capillary tube was directly visualized and imaged under POM. For SEM characterization, the glass tube was broken from one end and the sediment was dried in air at room temperature.

### Characterizations

Colloidal cuboids and their assemblies were imaged by using a Hitachi SU-70 Schottky field emission gun SEM at an operation voltage of 10 kV. TEM images and EDS maps were taken by using a JEOL 2100 FEG TEM at an operation voltage of 200 kV. XRD patterns were recorded with a Bruker Smart1000 (Bruker AXS Inc., USA) using Cu Kα radiation. FT-IR spectral measurement was performed on a Thermo Nicolet NEXUS 670 FTIR (32 scans) with a resolution of 4 cm^−1^. POM images were obtained by using a Nikon Eclipse Ti-S microscope with polarizer.

## Electronic supplementary material

Supplementary Information

## Data Availability

The authors declare that the data supporting the findings of this study are available within the article and its Supplementary Information files. Data is available from the corresponding authors upon reasonable request.
