# Supplementary material for: Synthesis and assembly of colloidal cuboids with tunable shape biaxiality
Source: Nat Commun. 2018 Oct 30;9:4513. doi: 10.1038/s41467-018-06975-8 (PMC6207716; doi:10.1038/s41467-018-06975-8)
Supplement: Supplementary file 1 — Supplementary Information [file 41467_2018_6975_MOESM1_ESM.pdf]

Synthesis and assembly of colloidal cuboids with tunable shape biaxiality

Yang *et al.*

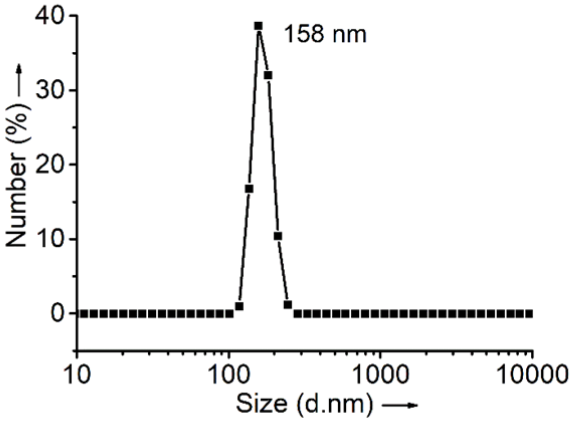

Supplementary Figure 1. Uniform nanosized water-in-oil emulsions. DLS measurement of emulsion droplets before adding NH_3. Pentanol viscosity: 4.0 cP, refractive index: 1.408.

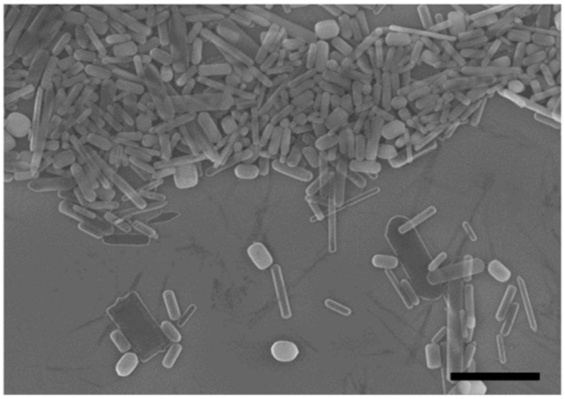

Supplementary Figure 2. Control experiment without *in situ* generation of $(NH_4)_2SO_4$. SEM image of non-uniform colloidal particles produced by directly adding $(NH_4)_2SO_4$ salt instead of *in situ* growth of $(NH_4)_2SO_4$. Scale bar, 2 µm.

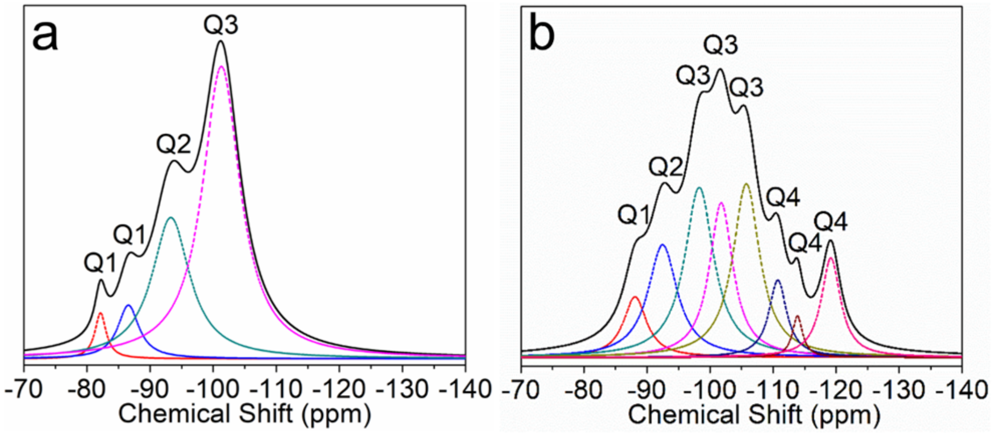

Supplementary Figure 3. The stability of the colloidal cuboids after washing with water. ^{29}Si NMR spectra of the colloidal cuboids (**a**) before and (**b**) after washing with water. The

NMR spectra showed that the Q4 (Si(OSi)4) portion of the cuboids after washing with water (about 40-min exposure to water) was higher than that of the ones without washing, indicating a higher degree of polymerization of silicate wall after washing. This is because washing with water lowers the pH of colloidal dispersion and promotes the condensation of silanols and redeposition of silicate anions.[1]

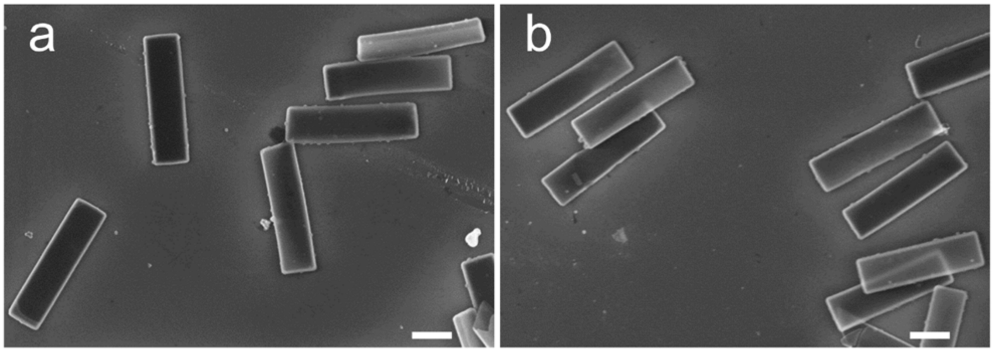

Supplementary Figure 4. The stability of the colloidal cuboids after washing with water. SEM images of the colloidal cuboids after washing with water stored in (**a**) ethanol for one year, and (**b**) water for ten days. Scale bars, 1 μm.

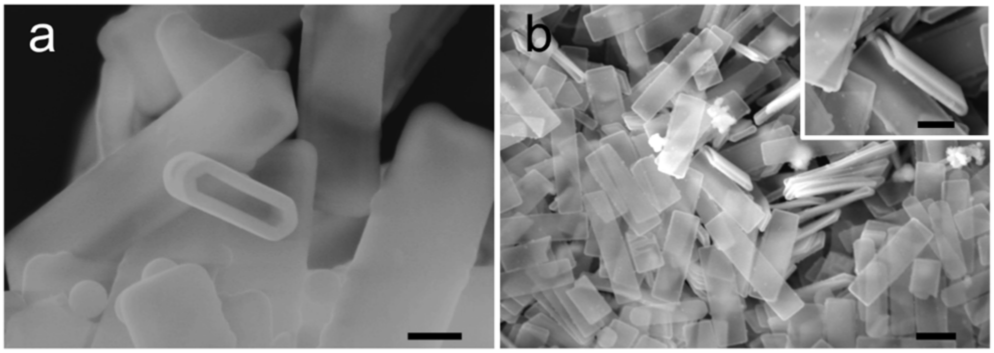

Supplementary Figure 5. Hollow silica shell of the colloidal cuboids. SEM images of cuboidal particles after removal of $(NH_4)_2SO_4$ cores via washing with water: silica shell with (**a**) T = 360 nm and Ti = 120 nm, and (**b**) T = 200 nm and Ti = 60 nm. Scale bars, 400 nm in **a**, 1 μm in **b**, and 500 nm in inset of **b**.

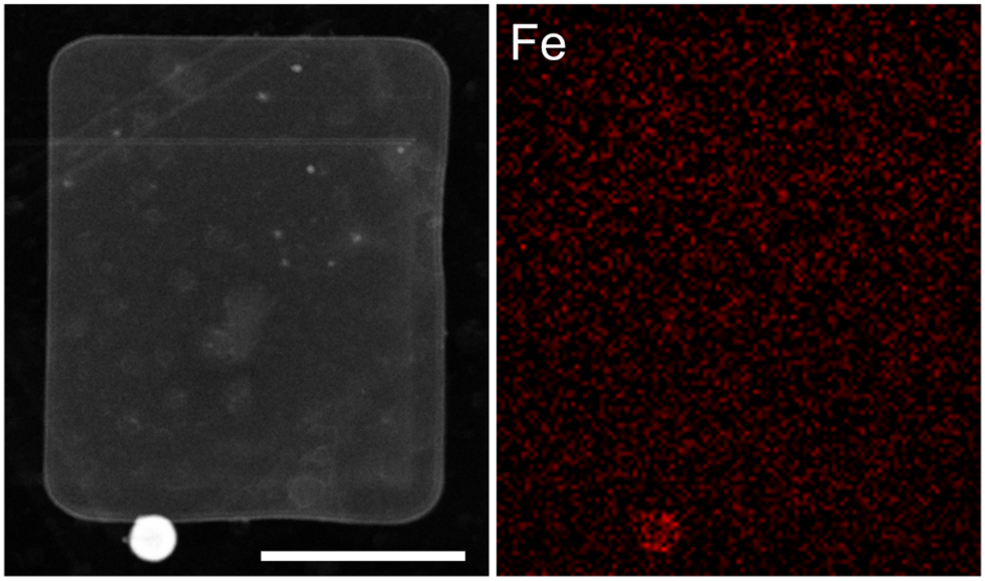

Supplementary Figure 6. Characterization of Fe distribution. Annular dark field TEM image and TEM-EDS map of Fe in a cuboid (L = 4800 nm, W = 3900 nm, T = 80 nm) and in the byproduct of an iron (III) hydroxide spherical particle. Scale bar, 2 μm. No obvious Fe could be detected in cuboids, while a strong Fe signal was observed in the byproduct of small spherical particles formed in the system. Moreover, we noticed in our experiment that the color of the reaction system immediately turned from white turbid to reddish brown upon the addition of ammonium aqueous solution, and reddish brown precipitates formed at the bottom of the vial after about 4 h. We, therefore, conclude that the iron (III) sulfate reacts with ammonium hydroxide to produce ammonium sulfate cuboidal crystals and reddish brown iron (III) hydroxide particles. Most of the iron (III) hydroxide particles precipitated and the rest small ones can be removed by centrifugation during the purification process of silica cuboids.

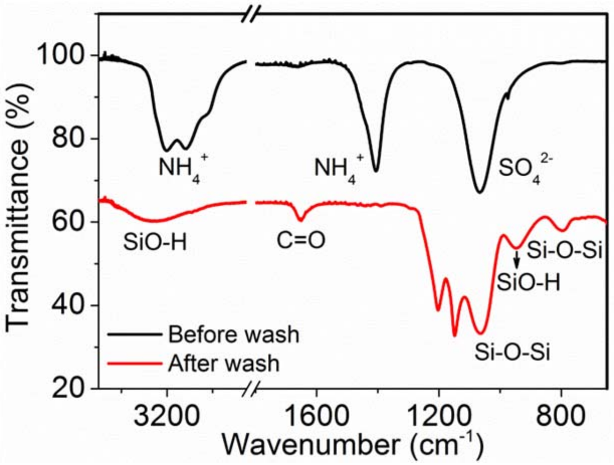

Supplementary Figure 7. Characterization of cuboidal colloid composition. FT-IR spectra of particles before and after washing with water.

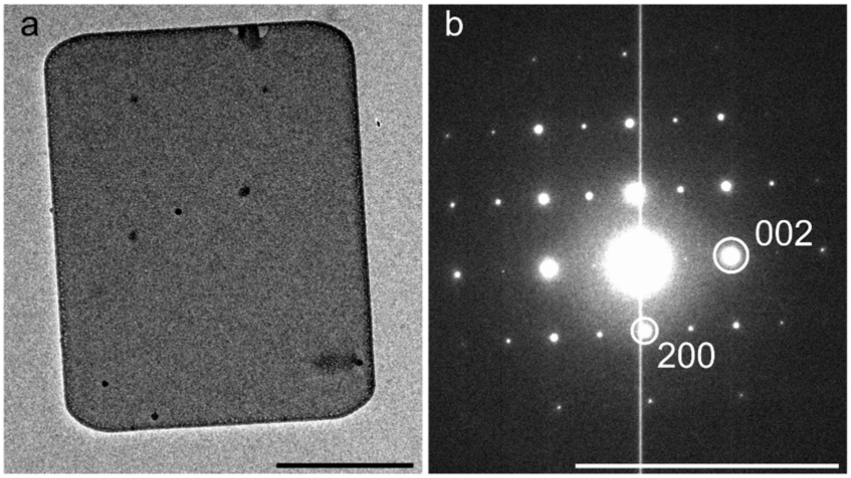

Supplementary Figure 8. Characterization of the single crystal core of colloidal cuboids. (**a**) TEM image of a cuboid, and (**b**) corresponding selected area electron diffraction pattern. Scale bars, 1 μm in **a** and 10 nm^{-1} in **b**.

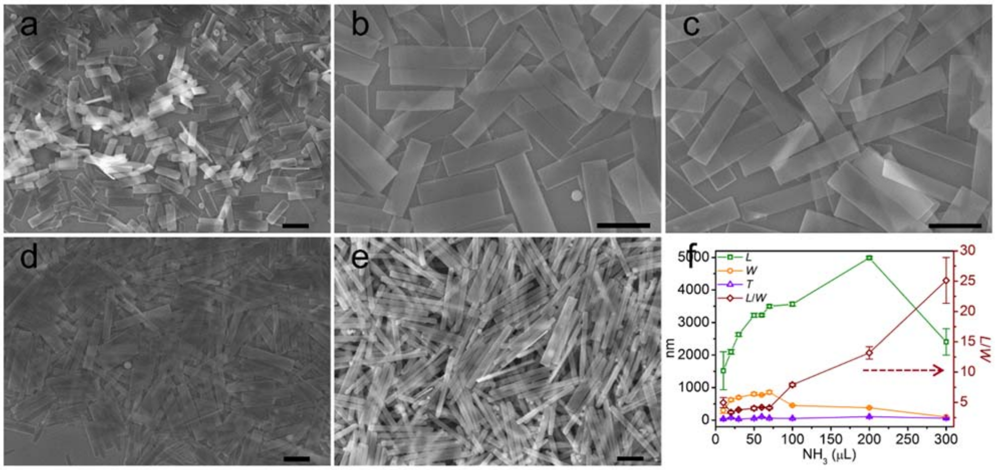

Supplementary Figure 9. NH$_3$ amount effect. SEM images of cuboidal colloids synthesized with the amount of NH$_3$ of (**a**) 20 μl, (**b**) 50 μl, (**c**) 70 μl, (**d**) 100 μl, and (**e**) 200 μl. (**f**) *L*, *W*, *T*, and *L*/*W* of particles as a function of the amount of NH$_3$. Error bars in **f** are the standard deviations by measuring 200 samples for each point. Scale bars, 2 μm.

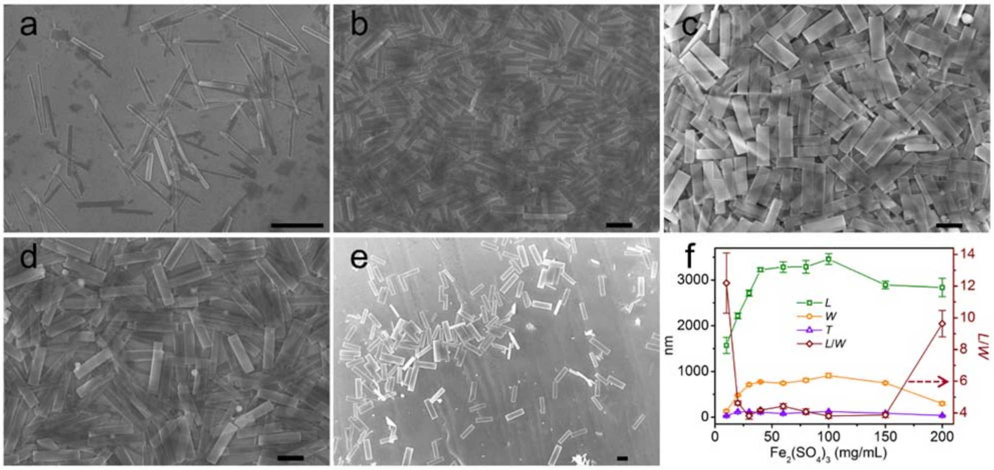

Supplementary Figure 10. $Fe_2(SO_4)_3$ concentration effect. SEM images of colloidal cuboids synthesized with the concentration of $Fe_2(SO_4)_3$ of (**a**) 10 mg ml^{-1}, (**b**) 20 mg ml^{-1}, (**c**) 30 mg ml^{-1}, (**d**) 60 mg ml^{-1}, and (**e**) 80 mg ml^{-1}. (**f**) L, W, T, and L/W of colloids as a function of the concentration of $Fe_2(SO_4)_3$. Error bars in **f** are the standard deviations by measuring 200 samples for each point. Scale bars, 2 μm. Note: the concentration of $Fe_2(SO_4)_3$ has to be in the range of 20–150 mg ml^{-1}, in order to obtain monodisperse colloidal cuboids.

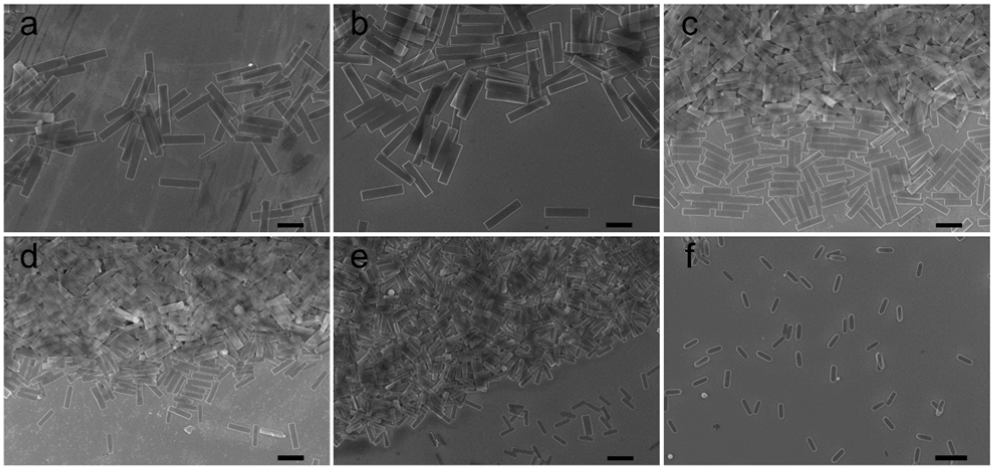

Supplementary Figure 11. Ethanol amount effect. SEM images of colloidal cuboids synthesized with the amount of ethanol of (**a**) 100 μl, (**b**) 300 μl, (**c**) 700 μl, (**d**) 900 μl, (**e**) 1000 μl, and (**f**) 1500 μl. Scale bars, 2 μm.

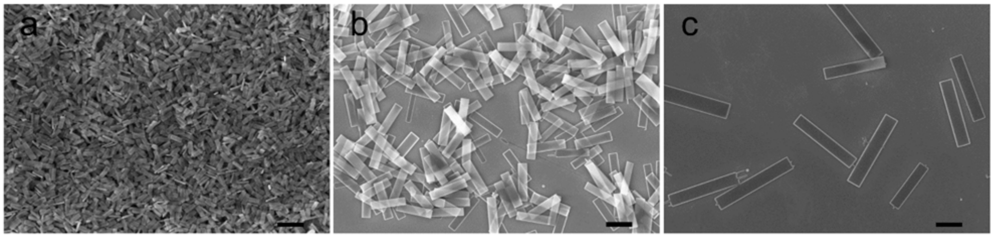

Supplementary Figure 12. PVP molecular weight effect. SEM images of colloidal cuboids synthesized by PVP with the molecular weight of (**a**) 10, (**b**) 29, and (**c**) 55 kg mol^{-1}. Scale bars, 2 μm.

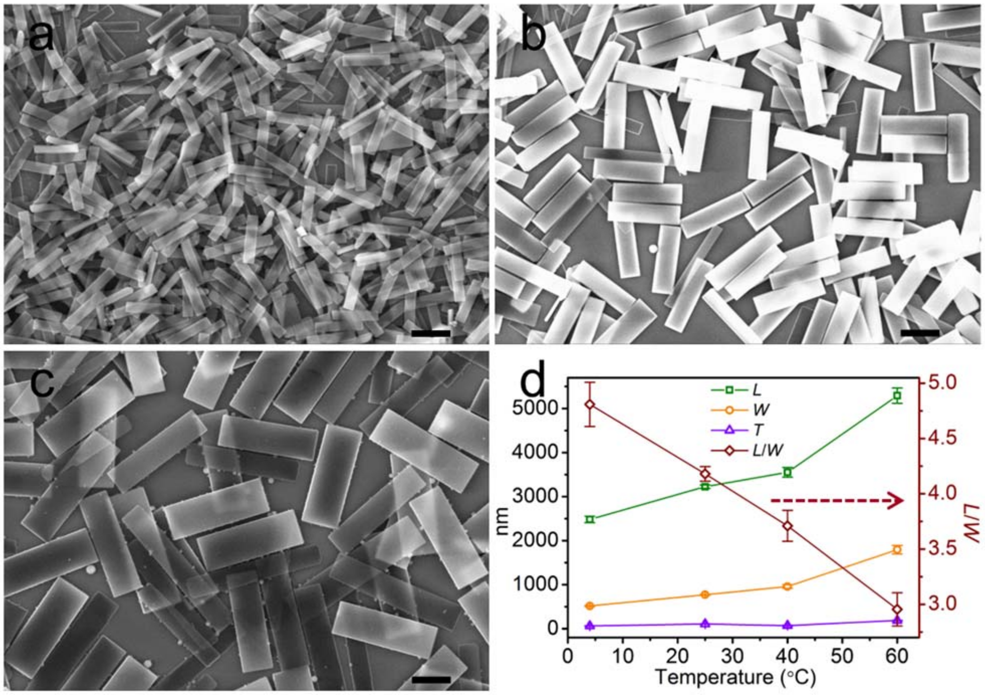

Supplementary Figure 13. Temperature effect. SEM images of colloidal cuboids synthesized at temperature (**a**) 4 °C, (**b**) 40 °C, and (**c**) 60 °C. (**d**) *L*, *W*, *T*, and *L*/*W* of colloidal cuboids as a function of temperature. Error bars in **d** are the standard deviations by measuring 200 samples for each point. Scale bars, 2 μm. The shape and size of colloidal cuboids could be controlled by varying reaction temperature. Higher temperature led to larger colloidal cuboids, but with smaller *L*/*W*.

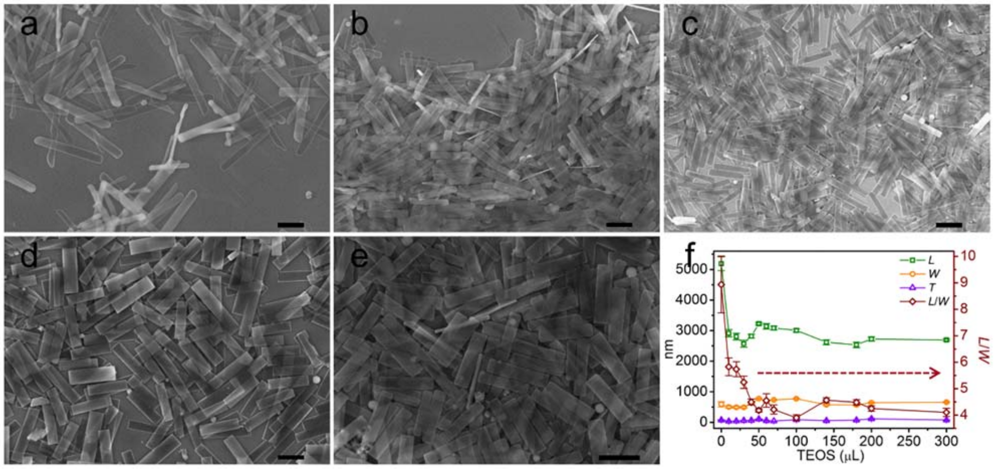

Supplementary Figure 14. TEOS amount effect. SEM images of colloidal cuboids synthesized with the amount of TEOS of (**a**) 0 μl, (**b**) 10 μl, (**c**) 20 μl, (**d**) 100 μl, and (**e**) 180

μl. (**f**) *L*, *W*, *T*, and *L*/*W* of colloidal cuboids as a function of the amount of TEOS. Error bars in **f** are the standard deviations by measuring 200 samples for each point. Scale bars, 2 μm. Note: to avoid the overgrowth of $(NH_4)_2SO_4$ crystals, the amount of TEOS had to be above 10 μl, in order to produce monodisperse colloidal cuboids.

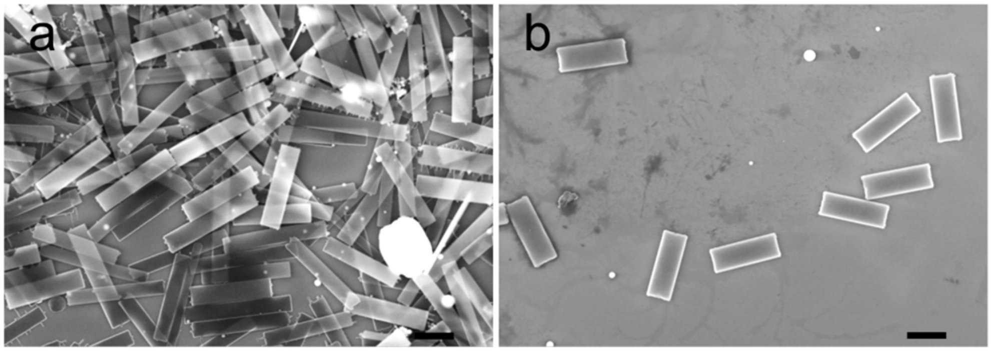

Supplementary Figure 15. Cation effect. Representative SEM images of colloidal cuboids with the additive of (**a**) $FeSO_4$ (46 mg ml^{-1}, 140 μl) and (**b**) $Ce_2(SO_4)_3$ (57 mg ml^{-1}, 140 μl). Scale bars, 2 μm.

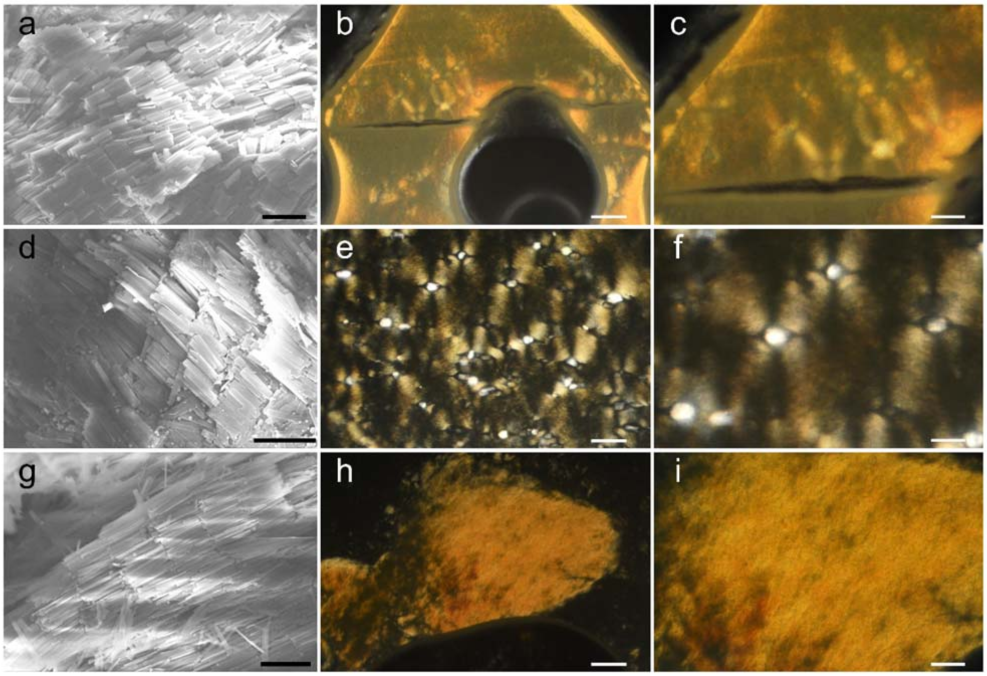

Supplementary Figure 16. SmAu phases of colloidal cuboids with θ > 0.05. Representative (**a**,**d**,**g**) SEM images, and (**b**,**e**,**h**) large-area and (**c**,**f**,**i**) enlarged POM images of colloidal cuboids in planar capillary of SmAu phases. SmAu phases assembled from colloidal cuboids of (**a**) $\theta = 0.20$, (**b**,**c**) $\theta = 0.28$, (**d**) $\theta = 0.15$, (**e**,**f**) $\theta = 0.31$, (**g**) $\theta = 0.07$, and (**h**,**i**) $\theta = 0.28$. Scale bars, 5 μm in **a**,**d**,**g**, 100 μm in **b**,**e**,**h**, 50 μm in **c**,**f**,**i**.

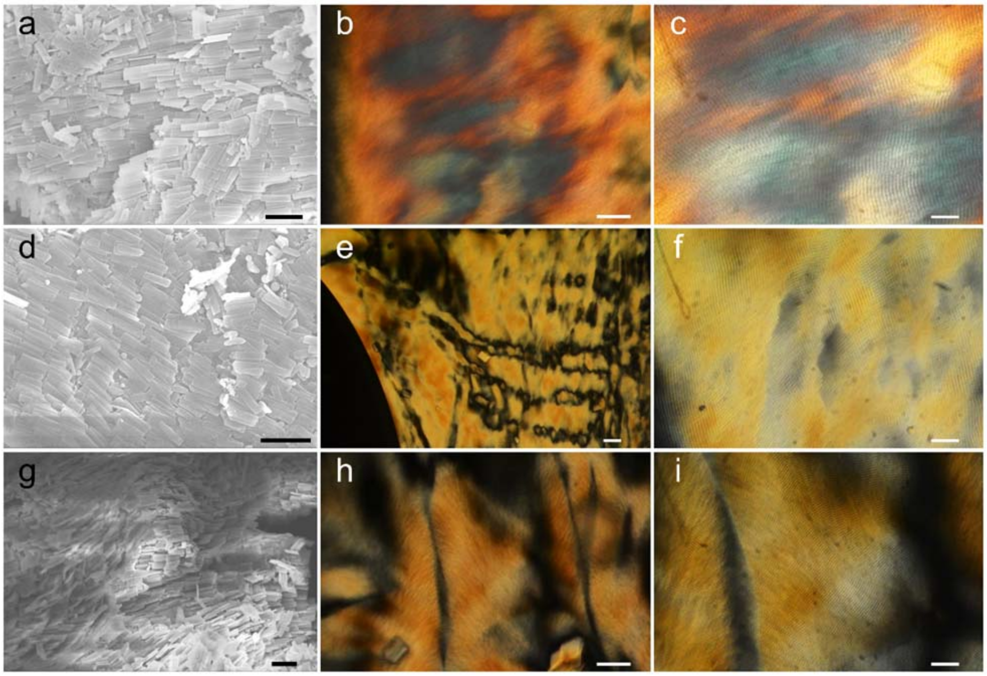

Supplementary Figure 17. SmAb phases of colloidal cuboids with –0.25 < θ ≤ 0.05. Representative (**a**,**d**,**g**) SEM images, and (**b**,**e**,**h**) large-area and (**c**,**f**,**i**) enlarged POM images of colloidal cuboids in planar capillary of SmAb phases. SmAb phases assembled from colloidal cuboids of (**a**) θ = –0.16, (**b**,**c**) θ = –0.04, (**d**) θ = –0.19, (**e**,**f**) θ = –0.14, (**g**) θ = –0.16, and (**h**,**i**) θ = 0.05. Scale bars, 5 μm in **a**,**d**,**g**, 50 μm in **b**,**e**,**h**, 20 μm in **c**,**f**,**i**.

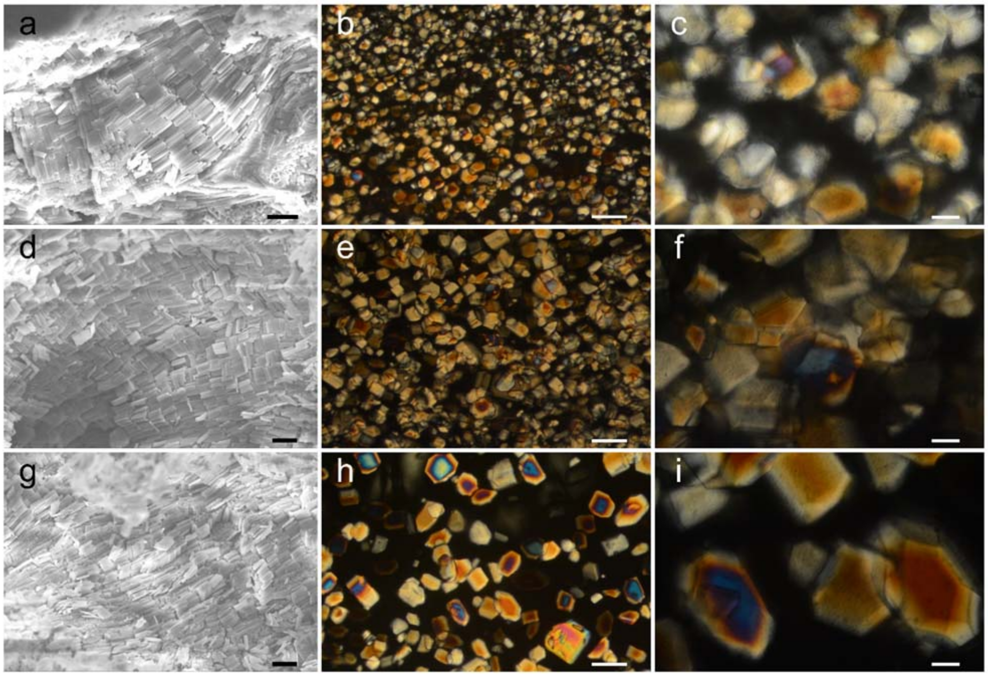

Supplementary Figure 18. Crystal B phases of colloidal cuboids with –0.25 < θ ≤ 0.05. Representative (**a**,**d**,**g**) SEM images, and (**b**,**e**,**h**) large-area and (**c**,**f**,**i**) enlarged POM images

of colloidal cuboids in planar capillary of crystal B phases. Crystal B phases assembled from colloidal cuboids of (**a**) $\theta = -0.11$, (**b**,**c**) $\theta = -0.09$, (**d**) $\theta = -0.16$, (**e**,**f**) $\theta = -0.03$, (**g**) $\theta = -0.16$, and (**h**,**i**) $\theta = 0$. Scale bars, 5 μm in **a**,**d**,**g**, 100 μm in **b**,**e**,**h**, 20 μm in **c**,**f**,**i**.

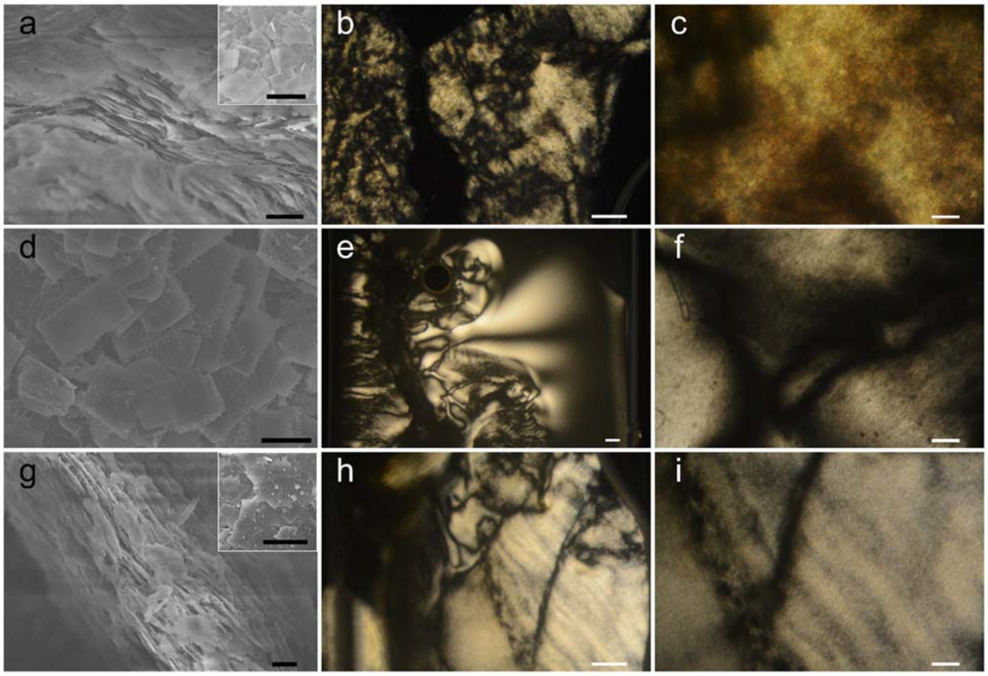

Supplementary Figure 19. N_{-} phases of colloidal cuboids with $-0.65 < \theta \leq -0.25$. Representative (**a**,**d**,**g**) SEM images, and (**b**,**e**,**h**) large-area and (**c**,**f**,**i**) enlarged POM images of colloidal cuboids in planar capillary of N_{-} phases. N_{-} phases assembled from colloidal cuboids of (**a**) $\theta = -0.51$, (**b**,**c**) $\theta = -0.51$, (**d**) $\theta = -0.54$, (**e**,**f**) $\theta = -0.37$, (**g**) $\theta = -0.56$, and (**h**,**i**) $\theta = -0.38$. Scale bars, 5 μm in **a**,**d**,**g**, 10 μm in insets of **a**,**g**, 100 μm in **b**,**e**,**h**, 20 μm in **c**,**f**,**i**.

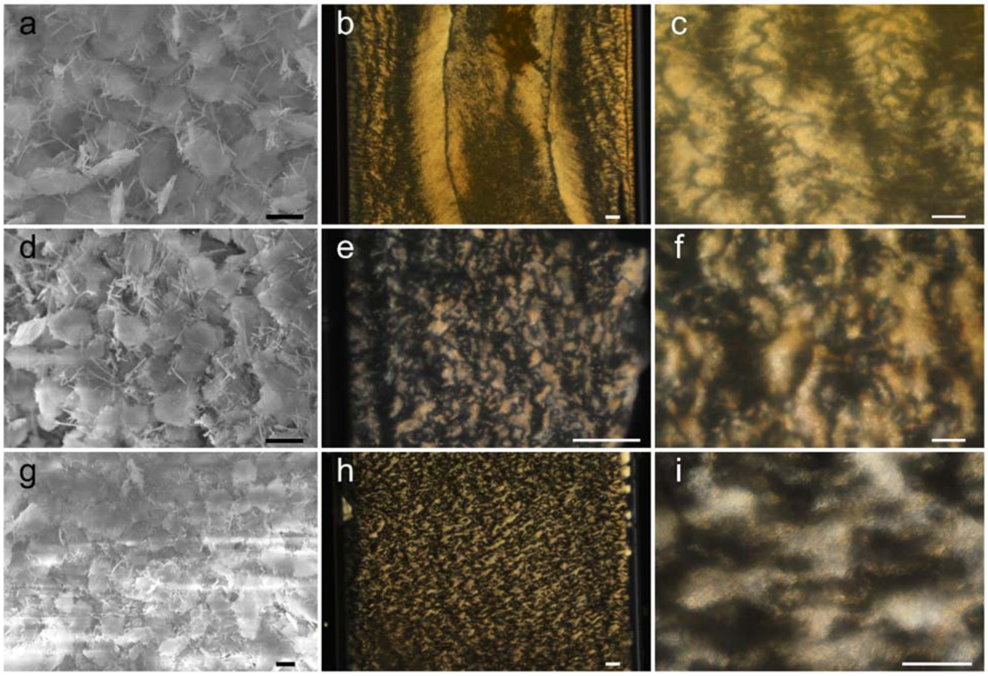

Supplementary Figure 20. Col phases of colloidal cuboids with $\theta \leq -0.65$. Representative (**a**,**d**,**g**) SEM images, and (**b**,**e**,**h**) large-area and (**c**,**f**,**i**) enlarged POM images of colloidal cuboids in planar capillary of Col. Col phases assembled from colloidal cuboids of (**a**) $\theta = -0.85$, (**b**,**c**) $\theta = -0.90$, (**d**) $\theta = -0.67$, (**e**,**f**) $\theta = -0.92$, (**g**) $\theta = -0.84$, and (**h**,**i**) $\theta = -0.78$. Scale bars, 5 μm in **a**,**d**,**g**, 100 μm in **b**,**e**,**h**, 50 μm in **c**,**f**,**i**.

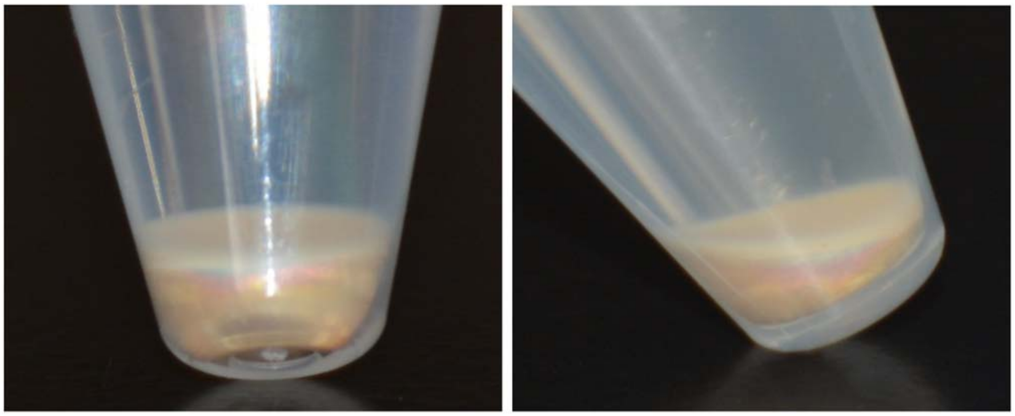

Supplementary Figure 21. Photonic crystal. Digital image of colloidal cuboids in a centrifuge tube, after sedimentation. Iridescent colors can be observed, depending on the volume fraction gradient of colloidal cuboids.

Supplementary References

1. Chen, L., Horiuchi, T., Mori, T. & Maeda, K. Postsynthesis Hydrothermal Restructuring of M41S Mesoporous Molecular Sieves in Water. *J. Phys. Chem. B* **103**, 1216-1222, (1999).
